# Genetic, familial and environmental correlates of asthma among early adolescents in Sri Lanka: a case control study

**DOI:** 10.1186/s40413-015-0068-x

**Published:** 2015-06-16

**Authors:** Manjula Nishanthi Danansuriya, Lalini C Rajapaksa, Anura Weerasinghe

**Affiliations:** Adolescent Health Unit Family Health Bureau Ministry of Health 231, De Saram Place, Colombo 10, Sri Lanka; Department of Community Medicine, Faculty of Medicine, University of Colombo, Colombo, Sri Lanka; Medicine and Immunology Dr Neville Fernando Teaching Hospital, South Asian Institute of Medicine and Technology, Malabe, Sri Lanka

**Keywords:** Asthma, Atopy, Adolescents, Sri Lanka, Skin prick test

## Abstract

**Background:**

Despite advances in management, the mortality and morbidity due to asthma are increasing globally. Identification of specific correlates in the local context is useful in disease management. The objective of this study was to estimate the prevalence and to describe selected correlates of asthma among12-14 year old school children in a district in Sri Lanka.

**Method:**

A school based cross-sectional study was conducted using a self administered questionnaire. Multi-staged stratified cluster sampling was used to select 42 classes in grades 7, 8 and 9. “Current asthma” (CA)(case) was defined as ‘having Physician Diagnosed Asthma (PDA) and having had wheezing during the previous 12 months’. For each case, two healthy controls were selected from the same class to assess correlates. Information on correlates was collected by trained field midwives during home visits. Backward stepwise logistic regression model was used for analysis of correlates. Skin Prick Testing was carried out among asthmatics together with their healthy siblings using standard extracts of House Dust Mite (HDM), cockroach and Blomia. Ethical clearance was obtained from Ethical Review Committee, Faculty of Medicine, Colombo.

**Results:**

Out of 1483 subjects participated, 753 were females (50.8%). The prevalence rates for current wheezing (CW), ever wheezing (EW), current asthma (CA), and physician diagnosed asthma (PDA) were 16.7%, 19.4%, 10.7% and 14.5% respectively. A total of 158 CA cases were identified. Information on correlates of asthma was collected for 145 CA cases (97.9%) and for 285 controls (96.6%). The unconfounded predictors of having CA among adolescents in the present sample were; only child in the family (OR = 4.2, 95% CI: 1.7-9.9); first born of the family (OR = 2.6 95% CI: 1.3-5.2); presence of allergic rhinitis (OR = 2.7, 95% CI: 1.6-4.6); family history of asthma (OR = 1.8, 95% CI: 1.1-3.2); family history of allergic rhinitis (OR = 1.9, 95% CI: 1.1-3.2); family history of eczema (OR = 1.8, 95% CI: 1.0-3.2). Higher risk of sensitization to cockroach, HDM and Blomia was seen among asthmatics compared to healthy siblings.

**Conclusion:**

A significant proportion of students reported to have asthma. Atopy and other genetic and environmental correlates should be considered as important correlates in asthma management among early adolescents in Sri Lanka.

## Background

Asthma is a syndrome characterized by airflow obstruction that varies spontaneously and with treatment [1]. Asthma is considered as the most common chronic illness in childhood, and despite advances in management; asthma morbidity and mortality are increasing globally [2,3]. The International Study on Asthma and Allergies in Childhood (ISAAC) revealed increasing prevalence of asthma in children and adolescents ranging from 0.8% to 32.6% [4,5].

The causality of asthma has been identified as a priority area in the field of asthma research [6]. It is presumed to be multi factorial [7]. The presence of family history of atopy, allergens, exposure to tobacco smoke, irritants and air pollution are considered as significant risk factors [2].

Asthma is a public health problem in Sri Lanka [8,9]. The prevalence of asthma among 5–11 year old children varies between 13% -25% [10,11] and asthma has been recognized as a major cause for school absence among primary school children [12]. The disease burden and risk factors for asthma in childhood have been studied in detail [10,11,13-16]. Information on impact of asthma on adolescents in Sri Lankan context is limited.

Adolescence is a period of transition with physical, mental and social changes. Not only the disease of asthma itself but its management also affects adolescents in specific ways, different to children and adults. Therefore asthma in adolescence should be given a special consideration and need to be studied extensively to support better clinical management. This is a part of a larger study which looked in to asthma burden, correlates and effects of asthma on the quality of life among early adolescents in an industrially developing setting like Sri Lanka.

The aims of the present study are to provide estimates on the prevalence of asthma and wheezing and to describe selected familial, genetic and environmental correlates of asthma among 12–14 year old school children in a selected district in Sri Lanka.

## Methods

The study was conducted in Gampaha district in the Western Province of Sri Lanka. The selected district ranges from highly urbanized, semi urbanized to rural areas and consists of a population of school children with diverse socioeconomic and ethnic composition. Gampaha is the second most populated district in Sri Lanka with a population of 2,294,641 as per the latest 2012 census [17]. The particular district was selected purposively considering above factors and the feasibility of data collection.

School children in grades 7, 8 and 9 in Sinhala medium schools were included. Physically or mentally handicapped children were excluded. Sample size for the prevalence study was calculated based on an expected prevalence of 20% and a precision level of 5%. The multi-staged stratified cluster sampling method was used. The original study which assessed prevalence, correlates of asthma among adolescents and their quality of life, employed a sample of 1512 students. The sample consists of 42 clusters and a cluster represented a class with 36 students. The students were given a self administered questionnaire with the questions on asthma and wheezing prepared based on the ISAAC Tool. Additional visits to the schools were made in instances where absentees exceeded 15% on the day of data collection.

Case control methodology was used to examine the correlates of asthma. Sample size for case control study was calculated based on the exposure as positive family history of asthma assuming 80% power with the use of 2-sided tests and an alpha level of 0.05. The non exposure rate for the family history of asthma was predicted to be 2% based on the adjusted odds ratio of 3.8 [14]. With 2:1 control matching, the number of cases and controls were 151 and 302 respectively.

Students reported to have Physician Diagnosed Asthma (PDA) and having had asthma symptoms (wheezing) during the previous 12 months for the self administered questionnaire, were categorized as Current Asthma (CA) and were considered as cases (n = 158) for the case control component. Students living outside the selected district and/or staying in a hostel/orphanage were excluded due to difficulties in collecting information.

For each identified student with Current Asthma (case), two “healthy” controls were selected from the same class according to the student’s self-response. We included a question inquiring presence of any chronic illness requiring long term medication or clinic follow up during the preceding six months period. Students answered negatively, were categorized as “healthy”. The two “healthy” students placed in the class register next to the “case” were taken as two controls. The student’s self-reported “healthy” and “asthma” status were further verified with the parent/guardian during home visits. We trained Public Health Midwives (PHM) as data collectors with a locally developed, pre tested interviewer administered “Home visit report” to collect information on correlates on asthma of adolescents during home visits.

Working definitions for selected correlates for data collection were constructed. Low birth weight was defined as a birth weight less than 2500 grams as recorded in the child health development record. Gestational period was determined as pre term, term and post term based on the records in the child health developmental record. Exposure to traffic was assessed by the proximity of the permanent residence to the closest main road. Houses situated closer to the main road (with heavy vehicular traffic) were considered as exposed to traffic. Presence of more than two persons in the student’s bedroom was considered as exposure to bedroom overcrowding [18]. Exposure to smoke due to biomass combustion was considered “positive” if the principal source of fuel was firewood and the cooking place/hearth was situated inside the house and absence of a chimney above the cooking place/hearth [19].

Exposure to irritant smoke (mosquito coils/joysticks) was taken as positive if those were being used regularly (more than 3–4 days a week) in the house. If the respondent described presence of cockroaches as “a few” or “a lot”, it was considered as positive exposure to cockroaches. With regard to cockroaches, the presence or absence of cockroaches was considered as more important than the number, therefore it was taken as exposed even if there were “a few” cockroaches according to the informant. Positive current exposure to pets was defined according to the presence of either cat or a dog at home at present. The presence of dampness in the bedroom was considered as a proxy indicator on exposure to mould in bedroom. The PHMs were asked to observe the place/room where the student used to sleep and inspect for the presence of a leaking roof/damp walls/damaged walls or plaster of wall/visible mould growth on wall/roof. If any of the above were present, they were taken as positive exposure to mould [18].

Biological parents and siblings were regarded as the immediate family in assessing the family history. Family history of rhinitis was defined by a positive answer to the question “Do you have any family member/s suffering from runny or stuffy nose, itchy red eyes with sneezing especially in the morning?” A family history of other allergies was considered positive in the presence of a family member with allergy towards drugs/food/or any other thing. Any passive exposure to tobacco smoke at present or during last one year period was considered as current exposure to tobacco smoke.

Female sex, having more than one child, later parity, term birth, appropriate birth weight, negative exposure to risk factors was taken as reference category in univariate and multivariate analysis.

Odds ratios were used for the assessment of unadjusted associations among categorical variables in the univariate analysis. The multivariate analysis was carried out to determine the best predictors of current asthma using a logistic regression model (backward stepwise). The dependant variable was coded as 0 and 1 for controls and current asthma cases respectively. All independent factors were included as categorical variables. The correlates that have been identified as predictors/risk factors by the previous researchers were entered to the model irrespective of their level of significance, demonstrated in the univariate analysis.

Variables entered to the model included, sex of the student; being the only child, being the first born of the family, presence of low birth weight, born as a pre term baby, having breast fed exclusively for 4 months, presence of allergic rhinitis or other allergies, presence of overweight, exposure to traffic smoke, or bed room overcrowding, or bio mass combustion or irritant smoke; exposure to cockroaches, presence of visible mould in the bed room, presence of current or past exposure to pets, presence of family history of asthma, allergy, eczema or allergic rhinitis and presence of present or past exposure to tobacco smoke. For the regression model, probability was fixed for entry at 0.05 level and removed at 0.1 significance level. Final model included 403 cases (93.7%) and the chi square was 96.0 (p = 0.000).

The Skin Prick Testing (SPT) was done to identify causative allergens in the atopic individuals using three common allergens; cockroach (*blattella germanica*), house dust mite (*dermatophagoides pteronyssinus*) and storage mite (*blomia*) standardized extracts from Stallergenes (France). Histamine (0.1%) and physiologic saline were used as controls.

Inclusion criteria for skin prick testing were; CA cases with a healthy sibling aged + or – 5 years. If the particular sibling had current asthma or asthma ever, they were excluded. Students living in a children’s home/orphanage were also excluded. Paired *t* test and McNemar’s test for difference between related samples were used as appropriate for the analysis.

## Results and discussion

The present study was carried out as a part of larger study aimed to estimate the prevalence of asthma, identify its correlates and to describe its effect on the quality of life among 12–14 year old school children. Multi stage cluster sampling method was used to select the sample for the prevalence survey. This analysis aims to provide with evidence on asthma prevalence and the correlates among early adolescents relevant to a developing country like Sri Lanka in order to support better clinical management of asthma among adolescents.

A total of 1483 students were enrolled from 42 clusters (classes). Majority were Sinhalese (95.1%) due to the inclusion of only Sinhala medium schools and the predominant Sinhala population in the district. There were 753 females (50.8%). The mean age was 13.38 years (SD = 0.8). Of the students 84% (n = 1251) reported absence of a chronic disease (Healthy) while 15.5% (n = 232) answered affirmatively.

The self reported prevalence rates were current wheezing (CW), ever wheezing (EW), current asthma (CA) and physician diagnosed asthma (PDA) were 16.7% 19.4% 10.7% and 14.5% respectively (Table 1). Higher prevalence of CW, EW, CA and PDA was observed among males (p < 0.05). Nearly 11% of students had exercise induced wheezing (CI, 9.3-12.5). These estimates were based on the questions adopted from the ISAAC tool, designed for population-based research and has been validated locally [10,13,14,20-22]. The definition for CA was constructed according to literature and expert opinion. Gampaha can be considered as a district with satisfactory health facilities and it is unlikely that an adolescent living with asthma, in Gampaha district would not have a diagnosis by a physician. The reported CA prevalence is lower compared to Karunasekara et al. (2003) which showed an asthma prevalence of 23% and also to 17%, reported by Amarasekara et al. (2010) [10,11]. It is important to note that the differences in case definitions used, age groups selected and sample selection could have influenced the disparity in results. The CW prevalence rates are within the rates reported by other local studies including ISAAC –Phase III [22]. The CW prevalence falls within the rates reported by the international ISAAC study which ranged from 5.1% to 37.6% [23]. Regional countries have reported lower rates as 6.4% in India and 11.7% in Pakistan and 5.2% in Indonesia which again can be explained as differences in age group selected and methodology used [23].

**Table 1:** **The prevalence of asthma and wheezing**

	**N**	**Prevalence %**	**95% confidence interval**
Current wheezing (CW)	248	16.7	14.8-18.6
Ever wheezing (EW)	288	19.4	17.3-21.3
Current asthma (CA)	158	10.7	8.9-14.5
Physician diagnosed asthma (PDA)	214	14.5	12.8-16.4

**Figure 1:**
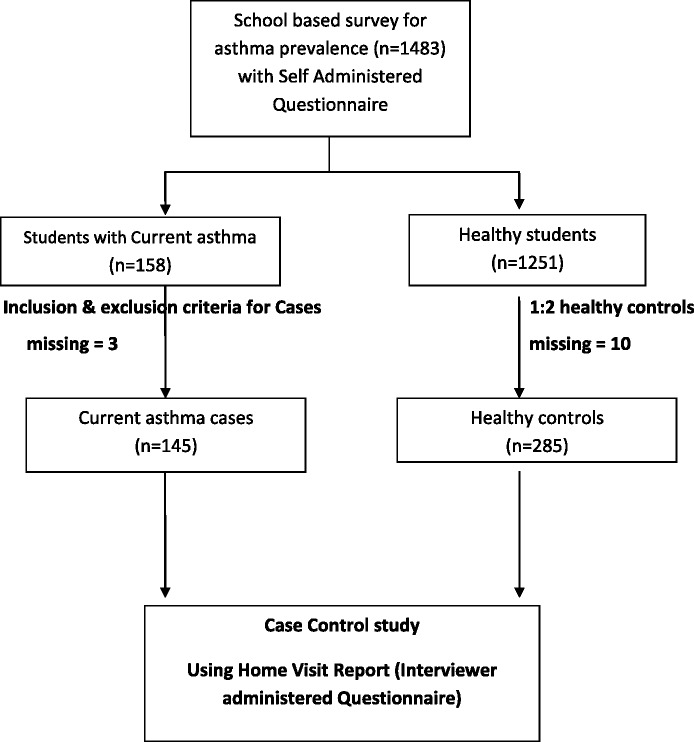
**Schematic diagram of the study.**

With regard to case control component of the study, of the 158 reported CA cases, eight students did not fulfil the selection criteria and two were excluded as their caregiver did not confirm the CA status. Midwives were able to collect information for 145cases only (1.8% missing). Of the 300 controls, five students were excluded as their “healthy” status did not corroborate with the caregiver and midwives were unable to collect information from ten healthy controls (3.3% missing) (Figure 1). The final analysis was based on 145 CA cases (97.9%) and 285 healthy controls (96.6%) (Table 2).

**Table 2:** **Socio demographic information of asthma cases and healthy controls**

**Variable**	**Case (N = 145)**		**Control (N = 285)**		**Unadjusted OR (95% CI)**
	**No**	**%**	**No**	**%**	
***Sex***					
Male	92	63.4	155	54.4	0.6 (0.4-1.0)
^$^Female	53	36.6	130	45.6	
***Number of children***					
One child	23	15.9	17	6.0	2.9* (1.5-5.7)
^$^More than one child	122	84.1	268	94.0	
***Birth order***					
First born	85	58.6	126	44.2	1.7* (1.1-2.6)
^$^Later born	60	41.3	159	55.7	
***Mother’s educational level***					
Up to secondary level	75	51.7	140	49.1	1.1 (0.7-1.6)
^)$^Above secondary	69	47.5	143	50.1	
Don’t know	1	0.6	2	0.7	

*significant at p < 0.05 level (2 tailed) ^$^-reference category.

The case control sample (n = 430) comprised of 178 females (41.8%) while our original sample had 50.8%. There were no significant differences among the ethnic composition between the two samples where 93% were Sinhalese in the sub sample compared to 95% in the original sample which was used to assess prevalence rates. In the subsample, 28.5% were living in urban areas compared to 25% in the original survey sample.

The univariate analysis revealed that born as the first child, only child, bed room overcrowding, exposure to cockroaches, presence of allergic rhinitis and other allergies, family history of asthma, allergic rhinitis and eczema were significantly associated with current asthma status of these early adolescents (Table 3). However, there was no significant association of male sex [24], low birth weight [25], pre term birth [26], exclusive breast feeding for 4 months [14], presence of overweight [27] exposure to traffic smoke or bed room overcrowding [14,28,29] or exposure to mould in the bed room with current asthma in the present univariate analysis. Past or present exposure to pets [30], family history of other allergies; present or past exposure to tobacco smoke [31] also failed to show significant relationships with current asthma status as per the univariate analysis. The observed variances could be due to true variances as per the global evidence which shows a diverse picture with regard to associations between asthma and exposures mentioned. It also could be due to variations in the operational definitions used, methods of data collection, recall bias, etc.

Of the variables selected for the regression model, six variables were identified as significant correlates for current asthma in this sample (Table 3). The unconfounded predictors for having current asthma among early adolescents in the present sample were; being the only child in the family (OR = 4.2, 95% CI: 1.7-9.9); being the first born (OR = 2.7% CI: 1.3-5.2); presence of allergic rhinitis (OR = 2.7, 95% CI: 1.6-4.6); positive family history of asthma (OR = 1.8, 95% CI: 1.1-3.2); positive family history of allergic rhinitis (OR = 1.9, 95% CI: 1.1-3.2); positive family history of eczema (OR = 1.8, 95% CI: 1.0-3.2).

**Table 3:** **Results of the logistic regression analysis of selected correlates of asthma**

**Variable**	**Unadjusted OR (95% CI)**	**Adjusted OR (95% CI)**
Being the only child	2.9 (1.5-5.7)	4.2 (1.7-9.9)
Being the first born	1.7 (1.1-2.6)	2.6 (1.3-5.2)
Presence of allergic rhinitis	2.7 1(1.7-4.1)	2.8 (1.6-4.6)
Presence of family history of asthma	2.5 (1.6-3.8)	1.8 (1.1-3.2)
Presence of family history of allergic rhinitis	2.1 (1.4-3.1)	1.9 (1.1-3.2)
Presence of family history of eczema	1.8(1.1-3.1)	1.8 (1.0-3.2)

*Significant at p < 0.05 level (2 tailed) N = 403 (93.7%).

The present study failed to reveal a significant association between CA and exposure to tobacco smoke as with other studies [13]. The true relationship between smoking and asthma may be masked by a change in behavior following diagnosis of asthma. Our findings did not show any increased risk of CA with exposure to traffic smoke or bio mass combustion or living in urban area. This may be due to that adolescents are likely to spend most of their time outside home environment. The association with pet exposure and asthma was difficult to explore using a cross sectional design and moreover, families with a history of atopy may avoid keeping pets [14,21].

Of the 158 reported CA cases, 94 were eligible for skin prick testing (SPT). Of them 60 students-sibling pairs participated for SPT (64% response rate). There were no statistically significant differences observed between the participants and non respondents in basic socio-demographic and other characteristics related to asthma or atopic conditions. Majority of the asthmatic cases (78.7%) were atopic compared to 16.4% among healthy siblings (p = 0.001). Cockroach was the commonest individual allergen, to which asthmatics were sensitive of (60.7%). It could be expected as cockroaches, are common in domestic environment, with a high probability of exposure to cockroach excreta. The present study found that students with current asthma were having 5.3 (95% CI: 2.2-12.6), 13 (95% CI: 3.8-54.7) and 11.5 (95% CI: 2.7-48.7), times higher risk of atopy towards cockroach, HDM and Blomia respectively compared to their healthy siblings. The wide confidence interval is a result of the relatively small sample size (Table 4). HDM and Cockroach have been identified as the commonest indoor allergens associated with CA in other local studies as well [32,33].

**Table 4:** **Paired analysis of atopy to allergens among cases and healthy sibling controls**

	**Controls (%)**			**Unadjusted odds ratio (95% CI)**
**Cases (%)**	**Positive**	**Negative**	**Total**	
**Blomia**				
Positive	4 (14.8)	23 (85.2)	27 (100.0)	OR = 11.5 (2.7- 48.7)
^$^Negative	2 (6.1)	31 (93.9)	33 (100.0)	
Total	6 (10.0)	54 (90.0)	60 (100.0)	
**House dust mite**				
Positive	3 (10.3)	26 (89.7)	29 (100.0)	OR = 13.0 (3.1-54.7)
^$^Negative	2 (6.5)	29 (93.5)	31 (100.0)	
Total	5 (8.3)	55 (91.7)	60 (100.0)	
**Cockroach**				
Positive	4 (11.1)	32 (88.9)	36 (100.0)	OR = 5.3 (2.2-12.7)
^$^Negative	6 (25.0)	18 (75.0)	24 (100.0)	
Total	10 (16.7)	50 (83.3)	60 (100.0)	

N = 60 *Significant at p < 0.05 level (2 tailed) ^$^-reference category.

## Conclusion

Asthma is found to be a common health problem among the 12–14 year old school children. The reported prevalence rates for current wheezing, current asthma and physician diagnosed asthma were 16.7%, 10.7% and 14.5% respectively. This study highlights atopy and other genetic and environmental correlates among early adolescents as important factors to be considered in asthma management.

## Abbreviations

CA: Current asthma
CW: Current wheezing
EW: Ever wheezing
HDM: House dust mite
PDA: Physician diagnosed asthma
SPT: Skin prick testing
ISAAC: International study on asthma and allergies in childhood
OR: Odds ratio
